# Simulation of Fatigue Fracture Detection of Bridge Steel Structures under Cyclic Loads

**DOI:** 10.1155/2022/8534824

**Published:** 2022-09-13

**Authors:** Dangfeng Yang, Lixiao Yao, Qi Pang

**Affiliations:** ^1^Xi'an University of Technology, Xi'an 710048, China; ^2^Northwest Engineering Corporation Limited, Power China, Xi'an 710065, China

## Abstract

Bridge steel structures are widely used in bridge construction with the advantages of light self-weight, convenient use, and good bridge span. Steel bridges are subjected to cyclic loading for a long time during their service period, and cyclic loading has a certain influence on their fatigue resistance performance. Fatigue is a phenomenon in which the structure is subjected to cyclic loading that generates cracks, expands continuously, and eventually leads to fracture of the member. The bridge steel structure under the repeated action of vehicle load and cyclic load is caused by microcracks and will expand with time, and the bridge deck system structure is prone to fatigue damage, so fatigue fracture detection has a great impact on the safe service life of steel bridges. In this paper, the fatigue design guidelines in the relevant codes and the bridge steel structure detection model are compared and analyzed, and a neural network-based fatigue fracture detection model for bridge steel structures under cyclic loading is proposed for the study of fatigue and corrosion interactions and fatigue and fracture of steel bridges under complex stress conditions. For this purpose, in the relevant experiments, experiments are designed to detect the fatigue fracture of bridge steel structures under different cyclic loads, and the experimental results prove the effectiveness of the proposed method.

## 1. Introduction

As an important part of the national transportation trunk line, most of the steel structure bridges hold the throat of highway and railroad transportation, and it is crucial to ensure their smooth and high-quality safe service. In recent years, China's economy has continued to develop at a high speed, and the traffic load on bridges has the important characteristics of heavy load, high speed, and high traffic volume [1–3]. To improve the fatigue resistance and fatigue life of steel bridges, scholars have carried out systematic research on the development of long-life structures and fatigue resistance design optimization methods from various perspectives, such as optimizing the matching of detailed structural parameters, improving the local structural force forms, improving the construction process, introducing high-performance concrete structural layers, and using high-performance materials [4–6].

**Figure 1:**
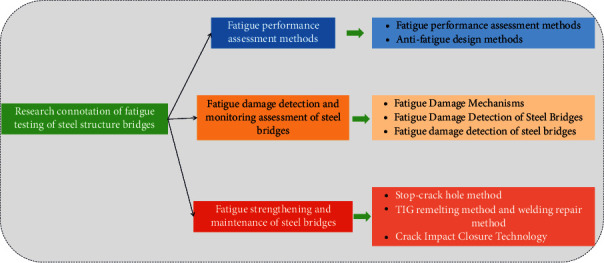
Research significance of fatigue fracture detection of bridge steel structures.

Steel bridges are an important development direction for bridge engineering because of their outstanding advantages such as light weight and high strength, large spanning capacity, easy factory manufacturing, and convenient assembly construction. However, engineering practice shows that fatigue and fracture are the key factors leading to the reduction of structural service performance and even catastrophic accidents, which seriously restrict the development and application of steel bridges [7]. At present, China's bridge engineering is in a critical transition period from construction-oriented to construction and maintenance-oriented, with the rapid development of new bridges and their expansion to offshore and difficult mountainous areas, and the safety and performance of steel bridges under extreme environmental and climatic conditions and events are in urgent need of research. At the same time, the aging, disease, and performance deterioration of steel bridges in service are becoming more and more prominent. The research significance of fatigue fracture detection of bridge steel structures is shown in Figure 1.

This paper presents an in-depth and systematic study of fatigue fracture of bridge steel structures under cyclic loading, now focusing on the main aspects of fatigue failure mechanism and resistance assessment methods, anti-fatigue design and construction techniques, environmental factors and their fatigue resistance effect mechanisms, fatigue crack identification and monitoring detection, and fatigue crack disposal and performance strengthening. The fatigue problem of steel bridges under cyclic loading is a research hotspot in the academic and engineering circles. Based on research on fatigue performance analysis and assessment theoretical methods, fatigue-resistant design and long-life structures, fatigue-resistant construction techniques, fatigue damage monitoring and fatigue micro-crack detection and identification, remaining fatigue life prediction, and fatigue performance strengthening, the construction of steel bridge whole-life cycle anti-fatigue technology is the future research [8–11].

The fatigue problem of steel bridges under cyclic loading is also gradually becoming serious. Bridge designers have a full understanding of the structural load carrying capacity, but do not have a full understanding of the fatigue load action leading to member or connection failure. As bridge collapse due to environmental erosion, excessive traffic flow, and vehicle overloading occurs, fatigue damage of bridges is increasingly receiving high attention from relevant departments and researchers. At present, there are three main fatigue design methods, the first is the infinite life design, which is a simplified design method, which requires the design stress of the structure lower than its fatigue limit stress value; *S*-*N* curve (stress-fatigue life curve) reflects the relationship between fatigue strength and fatigue life of the specimen under cyclic loading, according to the bridge design after knowing the stress amplitude *S*, according to the stress-fatigue. Based on the *S*-*N* curve, some scholars have proposed a safe life design to ensure that the member or connection can be used normally within the safe service life: damage tolerance design based on fracture mechanics to ensure that the damage will not be caused by crack expansion during the trial period by estimating its remaining life [12].

Current fatigue design codes around the world are mostly based on the latter two methods, and the codes of the UK, the USA, Japan, and Europe have the same safety determination guidelines and the classification of fatigue strength, while China's code for fatigue design provisions is relatively simple. In some cases, crack development will automatically stop at a certain level, and the crack will not continue to expand despite the continued action of cyclic loading. Calculating the size of the ultimate crack and the conditions of crack termination is the problem that fracture mechanics has been trying to solve in recent years [13–15]. The essence of fatigue damage is the development of cumulative damage under cyclic loading.

Fatigue damage of steel bridges under cyclic loading usually occurs at local hidden locations and is difficult to detect when the crack is small, but once it expands into a long crack, the structural safety risk and maintenance costs are significantly increased. At present, the traditional manual inspection and contact inspection are still the main means of steel bridge damage detection and monitoring, with low detection efficiency, high cost, easy to miss concealed cracks, and difficult to detect micro-cracks. To solve this problem, it is urgent to establish a real-time monitoring and evaluation system for fatigue damage of steel bridges under cyclic loading based on the properties of fatigue problems and the actual demand of its detection and monitoring, integrating the latest research results of nondestructive testing, and providing a scientific basis for the safety assessment and operation and maintenance decisions of steel bridges. Scholars have conducted a series of studies on the application of ultrasonic, acoustic emission, infrared thermography, image recognition, and other detection methods in the fatigue damage detection of steel bridges under cyclic loading [16].

The use of ultrasound and acoustic emission detection of internal defects and cracks is essentially the use of internal defects and cracks on the wave conduction of obscuration and reflection effect. Accurate identification of internal defects in longitudinal rib butt full fusion fillet welds of steel bridge panels was achieved by using ultrasonic phased array detection and identification method. An ultrasonic double probe penetration detection method was proposed for longitudinal cracks at the longitudinal rib weld of the top plate, and the detection method was tested and verified using prefabricated crack specimens. Piezoelectric ceramics (PZT) were embedded inside the steel UHPC composite structure, excitation was applied to them to generate ultrasonic waves, and the identification of local damage in the composite structure was achieved by ultrasonic signals. The key issues of nonlinear ultrasonic waves in weld detection were studied, and the fatigue damage characteristics of the weld in the acoustic signal were accurately extracted by combining Fourier transform and Hilbert–Huang transform. The expansion process of cracks in steel bridge panels was monitored by a combination of American Physical Acoustics (PAC), acoustic emission (AE) sensors, and PZT sensors, and the results showed that the technique can capture rich dynamic information of fatigue cracks in real time [17–19].

As a new technology in the ultrasonic inspection family, guided wave technology has the advantage of detecting all defects within the scan area in a single scan, while enabling defect quantification and localization. The array signal processing method based on singular value decomposition (SVD) extracts the damage dispersion signal in the guided wave, and the measured analysis of steel bridge panels shows that the proposed method can successfully extract the crack information from the measured signal with low signal-to-noise ratio and weak intensity. However, the multimodal characteristics of the guided wave and the complexity of the steel bridge panel structure will lead to a more cluttered guided wave signal, so the excitation and reception of the guided wave and the reasonable choice of signal processing methods are the keys to achieve accurate defect identification.

Steel bridges have been used more and more extensively in recent years, and the fatigue problem under cyclic loading has become more and more serious. However, bridge designers in China are not sufficiently aware of the failure of members or connections due to fatigue loading under cyclic loading. This paper first summarizes the fatigue design of steel bridges under cyclic loading, then compares and analyzes the fatigue design guidelines and methods under cyclic loading in the relevant codes, and finally proposes a neural network-based simulation for fatigue fracture detection of bridge steel structures under cyclic loading, which is used to analyze the fatigue fracture of steel bridges under cyclic loading in complex force cases. The comparison of test results shows that the model is simple and easy to implement, and the research results provide reference for the design application of steel bridges in practical engineering in China.

## 2. Related Work

### 2.1. Bridge Steel Structure Fatigue Fracture Detection

Since the damage of steel bridges caused by fatigue and fracture under cyclic loading is brittle, there is no ductile deformation process and obvious signs before the damage, and failure to detect fatigue cracking under cyclic loading in critical parts and dispose of them scientifically is the direct cause of serious diseases and catastrophic structural safety accidents of steel bridges. Scientific prediction of fatigue damage state and remaining life under cyclic loading is the basic premise to ensure high-quality service of steel bridges in service and to effectively avoid structural safety risks. However, for most of the steel bridges in China, especially those built in the early years, the fatigue load information under cyclic load and the actual fatigue damage degree and fatigue resistance under cyclic load of key structural details are missing during the service process; therefore, one of the main challenges in the study of fatigue under cyclic load of steel bridges in service is how to reconstruct the fatigue resistance of key structural details under the missing information [20–23].

The fatigue damage state under cyclic loading of structural details: The fatigue damage process under cyclic loading of structural details and the fatigue damage process under cyclic loading are cross-scale processes from microscopic to macroscopic, while the reconstruction process of the fatigue damage state under cyclic loading in the absence of information is a typical cross-scale inverse process [23–25]. The related key research contents include multi-physical parameter characterization method of fatigue damage state at key stages of cross-scale evolution of fatigue damage under cyclic loading; scale effect and nano-micro-scale structural test technology of fatigue resistance test under cyclic loading of key structural details; R&D of new test and inspection equipment for nondestructive and micro-destructive test of in-service structure with characterized physical parameters of damage state; multi-source feature information based on actual cyclic loading of key structural details; fatigue damage state inverse reconstruction method; and fatigue damage path determination and remaining life prediction method for in-service steel bridges under specific cyclic load.

In the whole fatigue life under cyclic loading action, microcrack sprouting extension life accounts for more than 50%; once the fatigue microcrack under cyclic loading action expands to macroscopic crack visible to the naked eye, it will rapidly expand to penetrating type grown-up crack. This leads to both problems of leakage detection prognosis and disposal timing. Due to the short time from stable expansion of macroscopic cracks to destabilization expansion, for some structures, the node or rod fracture caused by the missed detection of fatigue cracking under cyclic loading will directly trigger the catastrophic accident of brittle damage without aura in the structure; for fatigue tough structures under high resistance to cyclic loading, even if fatigue cracking under cyclic loading only reduces the service quality of the structure, the direct cost of reinforcement maintenance, the indirect cost due to the direct cost of reinforcement maintenance, and the indirect cost due to interference with traffic also increase exponentially with the increase of crack length. Due to the above characteristics, early identification of damage and timely disposal of fatigue cracking under cyclic loading are essential.

However, the identification of the early extension stage of fatigue cracking under cyclic loading is highly difficult and challenging, mainly because fatigue cracking under fine cyclic loading usually does not cause significant changes in the static and dynamic response of the structure, which is difficult to be accurately identified by traditional damage identification methods: the influence of significant deteriorating structural details such as initial manufacturing defects and corrosion and the fatigue resistance of the stressed member under cyclic loading. The distribution of these factors on the structure is strongly random, and the traditional manual inspection method has low efficiency, high cost, poor working conditions, and hidden cracks that are easy to miss, which makes it difficult to meet the actual demand of full-field inspection of the whole bridge; the fatigue problem of steel bridges under cyclic load has multiple structural details and multi-mode characteristics, and a considerable proportion of fatigue cracking under cyclic load is located in hidden parts, which is difficult to detect [26].

Based on the understanding of the basic properties of fatigue problems under cyclic loading, it is extremely necessary to construct an intelligent monitoring and identification system for fatigue damage under cyclic loading of steel bridges by introducing the latest advances in fatigue damage mechanisms under cyclic loading, fatigue resistance assessment methods under cyclic loading, testing and detection techniques, artificial intelligence, big data, and other fields. The system has already been explored and applied in the laboratory and in some steel bridge engineering practices. The related research will integrate the core research results of fatigue research under cyclic loading of steel bridges, directly support the goal of long life and high-quality service of steel bridges, and strongly promote the innovative research on fatigue problems under cyclic loading of steel bridges to a new stage of development.

This area is the key development direction for future steel bridges, and the main research contents in the next phase include fatigue damage characterization, and in-situ monitoring and identification methods for new structural systems and structural details under cyclic loading; real-time assessment and prediction systems for fatigue damage and cracking probability under cyclic loading based on multi-source information; real-time monitoring and measurement techniques for fatigue crack expansion under cyclic loading; micro-new technology of high precision nondestructive testing of fatigue cracks under cyclic loading; intelligent sensing technology and intelligent sensing system of fatigue damage under cyclic loading; theory and method of structural integrity assessment of steel bridges, etc. Infrared thermographic inspection technology is a technique to determine the damage on or near the surface of a structure by the difference between the target and the background temperature of the measurement point, which has the advantages of fast measurement speed, intuitive measurement results, and large detection area compared with ultrasonic inspection methods. Infrared thermal imaging NDT techniques can be classified as passive or active according to whether they depend on external heat source excitation. The results show that passive infrared thermography can detect fatigue cracks under cyclic load in steel structures under natural environmental conditions for the blocking effect of external heat transfer. With the development progress of computer vision technology, the apparent damage identification method based on computer vision has become a current research hotspot in the field of steel structure bridge damage identification.

The image acquisition device uses VGGNet-13 and VGG-FCN to classify the crack target sub-blocks. The crack geometry features of the real bridges are extracted by defining the crack pixel semantics. Various image segmentation techniques are used to locate and identify steel surfaces, and to convert steel structure rust images into HSV color space. The network input is a planar grayscale image of a CT specimen with scattered spots. The identification method of loose steel bridge bolts is proposed based on various machine vision techniques such as Fast-RCNN and perspective transformation [27–29]. The current computer vision technology has already begun to have rich visual information processing functions such as object recognition and tracking, scene reconstruction, image recovery, and measurement. Therefore, in addition to the above application scenarios, the technology can also be used for displacement measurement, modal parameter recognition, vehicle load recognition, etc., which has broad engineering application prospects. Vibration analysis-based local or global nondestructive testing techniques have received a lot of attention in recent years. Artificial neural network (ANN) is used as an intelligent localization and quantification tool for structural damage detection through vibration acceleration information. The damage localization was achieved by applying piezoelectric ceramic sheet on the surface of the plate and vibration excitation to it and using SLDV laser Doppler vibrometer to collect the vibration data excited at different frequencies, but the method has large environmental limitations and high cost, and how to optimize and streamline the testing process and apply it to the real bridge scenario will be the next stage of research.

This paper combines the latest development of intelligent detection technology and the basic properties of fatigue problems under cyclic loading of steel bridges, and initially summarizes the intelligent monitoring and identification methods of fatigue cracks under cyclic loading of steel bridges: (1) a systematic numerical simulation and experimental study of the application of ultrasonic guided wave in fatigue crack detection under cyclic loading of steel structures, and the results show the ultrasonic guided wave has good applicability in the detection of cracks in key-stressed members of steel bridges; (2) a smart sensor for fatigue crack nano-coating under cyclic loading was developed, theoretical research, numerical simulation, and experimental research were conducted on it, and the results show that the sensor has high sensitivity for small cracks; (3) a research was conducted on the fatigue crack recognition method based on computer vision under cyclic loading and the actual; the results show that the computer vision technology has high reliability in fatigue crack recognition under cyclic loading; (4) the deep learning theory is introduced into the pattern recognition of fatigue damage state under cyclic loading of the structure, which is used to mine and recognize the damage information in the time domain signal and image signal. The results show that the neural network system based on monitoring data and targeting damage pattern recognition has a broad application prospect in practical engineering.

### 2.2. Artificial Intelligence Technology

Visual defect detection aims to locate and identify defects in images, which is one of the classical tasks in computer vision, is the premise and foundation of many computer vision tasks, has important application value in the fields of automatic driving and video surveillance, and has received wide attention from researchers. With the rapid development of deep learning technology, defect detection has made great progress. The training phase includes data preprocessing, detection network, label assignment, and loss function calculation, and the testing phase uses the trained detector to generate detection results and postprocess the results [30, 31].

Monocular visual defect detection is the basis of visual defect detection, which aims to predict the location and class information of defects present in a single image. Since the success of deep convolutional neural networks for image classification tasks in 2012, researchers have started to experiment with deep convolutional neural networks for defect detection. Since then, deep learning-based defect detection has started to dominate the development of defect detection. The defect detection methods are divided into three categories: anchor box-based defect detection methods, anchor box-free defect detection methods, and end-to-end prediction defect detection methods. It should be noted that the end-to-end predictive defect detection method belongs to the defect detection method without anchor frames. Since the end-to-end predictive defect detection method does not require postprocessing operations and mostly uses a converter model to directly predict a detection result for each defect, it is a more concise detection architecture and will be introduced in detail as a separate category.

Anchor box-based defect detection method sets multiple rectangular boxes for each location in space to cover all defects present in the image as much as possible. Defect detection based on anchor boxes can be divided into two categories: two-stage defect detection methods and single-stage defect detection methods. The two-stage method first extracts *k* candidate detection windows of nonspecific categories and then further classifies and regresses these candidate detection windows to generate the final detection results. Unlike the two-stage method, the single-stage method directly classifies and regresses the anchor frames. In general, the two-stage method has higher detection accuracy, while the single-stage method has faster inference speed.

The two-stage defect detection method regional convolutional neural network series work is the most representative work of the two-stage defect detection method. R-CNN first uses selective search method to generate 2000 most likely defective candidate detection frames, then uses deep convolutional neural network to extract the depth features of these candidate detection frames, and finally uses support vector machine for classification and regression. This method was a great success at that time and substantially improved the accuracy of defect detection. Since the R-CNN extracts the depth features of each candidate frame separately, there is a problem of slow inference speed. To address this problem, the features of the whole image are extracted by feature sharing, and then the features corresponding to each candidate frame are converted into fixed-length features for subsequent SVM classification and regression using spatial pyramid pooling operation, which is referred to as SPP-Net. R-CNN and SPP-Net feature extraction and prediction (classification and regression) is a multi-stage process, which limits the performance of deep neural networks, and an improved work Fast R-CNN is proposed for R-CNN. Fast R-CNN first extracts the depth features of the whole image, then scales the features of the candidate detection frames to a fixed size using the region of interest pooling operation, and finally uses the fully connected layer for classification and regression.

The Fast R-CNN further unifies the generation of candidate windows with the classification and regression of candidate windows into a single network for joint learning. The RoI pooling operation can extract contextual information, and PS RoI can capture local information of defects. The features extracted by RoI pooling operation and PS RoI pooling operation are fused and used for subsequent classification and regression. The deformable RoI pooling operation can better characterize the deformation of defects, and the RoI align pooling operation can solve the feature mismatch problem caused by the quantization error of RoI pooling operation. Cascade R-CNN, a cascading defect detection architecture, cascades multiple Fast R-CNN head networks to further classify and regress the classification and regression results of the previous level in the current level. The cascade idea is used for candidate window generation. To cope with the variation of defect scales, the researchers proposed an image pyramid-based approach and a feature pyramid-based approach. The image pyramid-based approach uses images of different scales to detect defects at different scales, such as small-scale images to detect large-scale defects and large-scale images to detect small-scale defects. The image pyramid-based method requires the use of detection networks to detect multiple images of different scales, and the computational effort is relatively large. The feature pyramid-based method uses different layers within a single detection network to detect defects at different scales, which is relatively less computationally intensive.

## 3. Methods

### 3.1. Model Architecture

**Figure 2:**
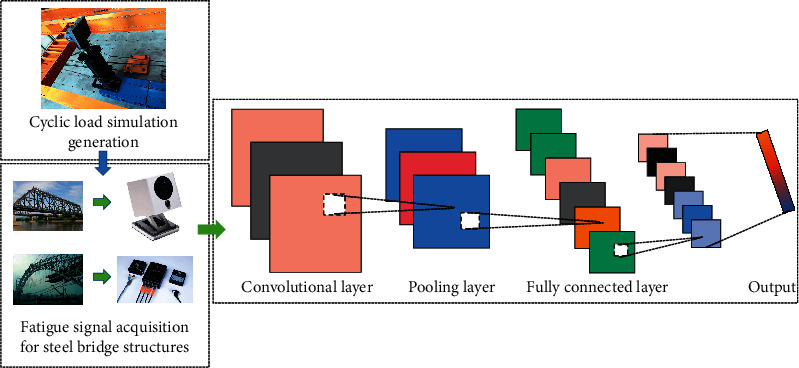
Model structure.

It is very important for fatigue fracture detection of bridge steel structures under cyclic loading. To improve the detection efficiency, using a convolutional neural network model based on deep learning to evaluate the corrosion level of the corrosion layer helps to avoid misjudgment due to human fatigue and inattentiveness. Using its efficient and automated feature extraction, this chapter proposes to establish a fatigue fracture detection model for bridge steel structures based on convolutional neural networks, as shown in Figure 2. The model can achieve high accuracy in distinguishing steel plates with different fatigue fracture levels, which provides a theoretical basis for subsequent practical applications. In addition, the parameters of the trained VGG-corrosion model will be saved and can be loaded directly as pretrained model weights based on the migration learning strategy in the subsequent practical applications, thus reducing the demand for training sample size in practical applications.

### 3.2. Steel Structure Relationship under Cyclic Loading

In this paper, the intra-temporal intrinsic structure theory is used. The cyclic intra-temporal intrinsic structure relationship can be expressed as

(1) $$\begin{array}{c} S_{ij}=\int_{0}^{Z_{D}}\;\rho \left(Z_{D}-Z\right)\frac{\partial e_{ij}^{\rho }}{\partial Z}dZ\text{,} \end{array}$$

where *S*_*ij*_ is the stress bias tensor, *e*_*ij*_^*ρ*^ is the plastic strain bias tensor, and *Z*_*D*_ is the skewed internal time scale.

(2) $$\begin{array}{c} dZ_{D}=\frac{||de_{ij}^{p}||}{f}\left(\xi \right)\text{,} \end{array}$$

*ξ* is the internal time metric, *ρ*(*Z*) is the core function, *f*(*ξ*) is the hardening function, and ||−|| denotes the parametric number in Euclidean space. For isotropic incompressible materials under hydrostatic pressure, there are elastic relations.

(3) $$\begin{array}{c} \sigma_{KK}=3K\varepsilon_{KK}\text{,} \end{array}$$

The total strain bias consists of both elastic and plastic components.

(4) $$\begin{array}{c} de_{ij}^{p}=de_{ij}-\frac{1}{2G}dS_{ij}\text{,} \end{array}$$

The relationship between strain tensor and strain bias and stress tensor and stress bias is

(5) $$\begin{array}{c} de_{ij}=d\varepsilon_{ij}-\frac{1}{3}d\varepsilon_{aa}\delta_{ij},dS_{ij}=d\sigma_{ij}-\frac{1}{3}d\sigma_{aa}\delta_{ij}\text{.} \end{array}$$

Substituting the differential equations into the incremental equation,

(6) $$\begin{array}{c} d\sigma_{ij}=2\hat{G}d\varepsilon_{ij}+\frac{3K-2\hat{G}}{3}d\varepsilon_{aa}\delta_{ij}+\frac{2\hat{G}}{\rho \left(0\right)}h_{ij}\left(Z_{D}\right)dZ_{D}\text{.} \end{array}$$

This is the tensor representation of the intrinsic structure relationship within the incremental form applied to the model in this paper.

(7) $$\begin{array}{c} \begin{array}{c} h_{ij}\left(Z_{D}\right)=\int_{0}^{Z_{D}}\;\hat{\rho }\left(Z_{D}-Z\right)\frac{\partial e_{ij}^{p}}{\partial Z}dZ,\hat{\rho }\left(Z_{D}-Z\right)=\frac{d\rho \left(Z_{D}-Z\right)}{dZ}\\ 2\hat{G}=\rho \left(0\right){\left\{1+\frac{\rho \left(0\right)}{2G}\right\}}^{-1} \end{array}\text{.} \end{array}$$

In this paper, the core function is taken as follows:

(8) $$\begin{array}{c} \rho \left(Z\right)=\overset{3}{\underset{r=1}{\sum }}\;R_{r}e^{-\beta_{r}Z}\text{.} \end{array}$$

The constant *R*_*r*_ has the dimension of stress, and *β*_*r*_ is a dimensionless quantity. The reinforcement function is taken as *f*(*ξ*) = 1, while *R*_*r*_ and *β*_*r*_ are obtained by curve fitting based on the material test curve.

### 3.3. Fatigue Time Delay Constraint

**Figure 3:**
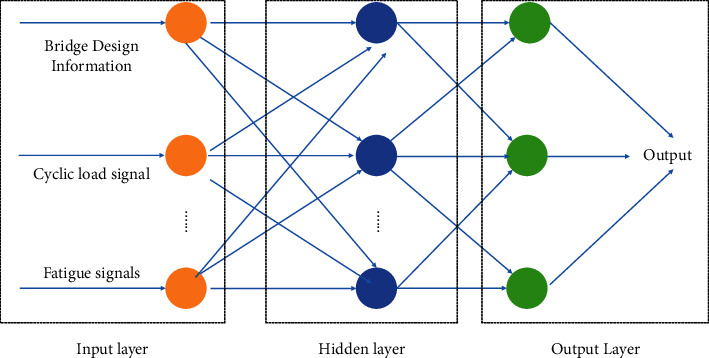
Neural network detection architecture.

The model light weighting under the time delay constraint is to use the inference time delay of the model as one of the constraints for model training and use the time delay as a direct indicator to guide the training process of the model. The neural network detection architecture is shown in Figure 3. The goal of this method is to minimize the loss function under the condition that the predefined latency constraint is satisfied as follows:

(9) $$\begin{array}{c} \min_{W}\;l\left(W\right),\tau \left(W\right)\leq T_{\text{budget}}\text{,} \end{array}$$

where *𝒲* denotes the weight tensor of all layers and *τ*(*𝒲*) denotes the actual inference delay of the network, which depends on the structure of the network model's weights *𝒲*. Compressing the network model affects the model's weights *W* and thus the model's inference delay *τ*(*𝒲*). *ℓ* is the loss function for a particular learning task. In deep learning, *ℓ* is usually a highly nonconvex function. The compression of the model is achieved by pruning, and as mentioned before, there are two different types of pruning techniques: structured and unstructured. Unstructured fine-grained pruning removes individual elements, while structured coarse-grained pruning deletes the regular structure of the DNN, such as the channels of the convolutional layer or even the whole convolutional layer. While both approaches are applicable here, this paper focuses specifically on the coarse-grained approach, which prunes channels or layers, because channel pruning is more effective on off-the-shelf DNN hardware platforms (e.g., GPUs) and more likely to achieve the goal of inference latency reduction, while the fine-grained approach requires specialized hardware architectures to be effective. Using a coarse-grained structured pruning approach, the optimization problem becomes finding the sparsity of each layer, i.e., the number of channels retained in each layer, such that the total inference latency of the model satisfies a given budget, i.e.,

(10) $$\begin{array}{c} \min_{W,s}\;l\left(W\right)\phi \left(w^{\left(u\right)}\right)\leq s^{\left(u\right)},u\in U\;\tau \left(W\right)\leq T_{\text{budget}}\text{,} \end{array}$$

where *s*^(*u*)^ corresponds to the sparsity bound of layer *u*. The inference delay *r* of the DNN can now be expressed as a function of *s*. *w*^(*u*)^ denotes the weight tensor of layer *u*. For a convolutional layer *u*, if it has *d*^(*u*)^ output channels and *c*^(*u*)^ input channels, the shape of *w*^(*u*)^ corresponding to this convolutional layer is *d*^(*u*)^ × *c*^(*u*)^ × *r*_*h*_^(*u*)^ × *r*_*w*_^(*u*)^; without loss of generality, we consider the fully connected layer as a special convolutional layer.

### 3.4. Bilinear Time Delay Detection Model

To evaluate the inference delay of the initial model as well as the model after compression, if the model is executed once completely each time to obtain the inference delay of the model, this will undoubtedly be very time-consuming during the experiment and greatly reduce the efficiency of the experiment. The key aspect of solving the equation is to obtain the inference delay *τ*(*𝒲*) of the model by the sparsity *s* of each layer of the model to determine whether it still satisfies the constraint, i.e., modeling the inference delay of the DNN model as a function of the sparsity of each layer. This step is particularly important because it provides an analytical form to characterize the delay. Existing DNN latency models are dedicated to specific hardware platforms, which requires a deep understanding of the hardware and cannot be ported across different hardware architectures. Latency prediction models can be constructed directly from measurements and computations on the hardware, while treating the hardware platform as a black box. This idea is like Net-Adapt, which builds latency models from hardware measurements. However, these models are like a huge nonminiature lookup table, which greatly reduces the efficiency. Therefore, the goal of this chapter is to construct a microscopic model to solve optimization problems using traditional gradient-based algorithms. The main idea of this paper is that the time delay model can be obtained by a data-driven approach. Let *r* be a differentiable function to approximate *τ*.

(11) $$\begin{array}{c} \hat{\tau }=\underset{f\in F}{\text{argmin}}E_{s}\left[{\left(f\left(s\right)-\tau \left(s\right)\right)}^{2}\right]\text{,} \end{array}$$

where *f* is the space consisting of all potential sparse models and their corresponding inferred time delays, and *E*_*s*_ is the expectation. To find a differentiable delay model, our intuition is that the time consumption of a layer in a DNN is influenced by the number of channels in its input and output feature mapping, which in turn corresponds to the sparsity of the current layer and the next layer, respectively. Thus, the time consumption of layer *j* can be obtained by modeling a function of the interaction between *s*_*j*_ and *s*_*j+1*_, where *s* denotes the sparsity of layer *j*. Based on this intuition, we use the following bilinear model to approximate the total network model time delay.

(12) $$\begin{array}{c} F≔\left\{f\left(s\right)=a_{0}+\overset{U\;|\;}{\underset{j=1}{\sum }}\;a_{j}s_{j}s_{j+1},a_{0},a_{1},\ldots ,a_{R1}\in R_{+}\right\}\text{,} \end{array}$$

where *s* is defined as the network output dimension, e.g., the number of classes in the classification task. The coefficients *a*_0_, *a*_1_,…, *a*_*ℜ*1_ are the variables that define this space. The rationale for using a bilinear structure is that the total number of arithmetic operations (multiplication and addition) during DNN inference for the layers defined by *s* is roughly in bilinear form. While other more complex models are possible, the bilinear model is simple and compact, and easy to train, and effectively avoids overfitting. For some specific DNN architectures, what happens if the time delay function cannot be essentially modeled in bilinear form. In this case, a neural network can be used to approximate the delay function, since a three-layer neural network can theoretically approximate any function. The constrained optimization in the equation only requires the delay model to be differentiable, so it still applies. To obtain *τ*, *s* is sampled from a uniform distribution and the actual inference latency on the target hardware platform is measured to obtain *τ*(*𝒲*). The stochastic gradient descent method is then used to solve the equation and obtain *τ*. For a given combination of network and hardware, the procedure is performed only once.

## 4. Experiments and Results

### 4.1. Experiment Setup

The software and hardware configurations used in this study are shown in Table 1. To study the ultra-low circumferential fatigue fracture behavior of steel bridges under horizontal two-directional earthquake, Q345qC, a bridge steel commonly used in Chinese steel bridge construction, was selected as the material, and two thick-walled steel bridges with stiffening ribs were designed and machined according to the design code for highway steel bridges. The two steel bridges are numbered *S*-1 and *S*-2, respectively.  Table 2 gives the values of structural geometry, including section width B and length *D*, structural height *h*_1_, stiffening mother plate thickness *t*, transverse spacer thickness *t*_*d*_, stiffening rib thickness *t*_*s*_, stiffening rib height *h*_*s*_, transverse spacer spacing a, longitudinal stiffening rib spacing *a*′, and section width and length directions divided by longitudinal stiffening ribs as *n*_*x*_ and *n*_*y*_, respectively. The stiffening ribs are connected to the stiffening mother plate by fillet welds, and the adjacent mother plate and the mother plate are connected to the base plate by full penetration welds. The basic mechanical properties of steel bridge steel Q345qC are shown in Table 3, including modulus of elasticity *E*, Poisson's ratio *μ*, yield strength *o*_*y*_, ultimate strength *o*_*u*_, and strain at break cy and elongation *A*. The values of these parameters were obtained from the material property test results of Q345qC steel.

**Figure 4:**
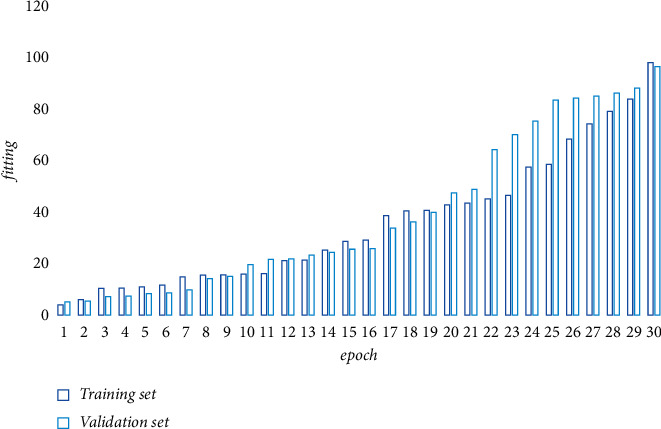
The training process loss convergence schematic.

**Figure 5:**
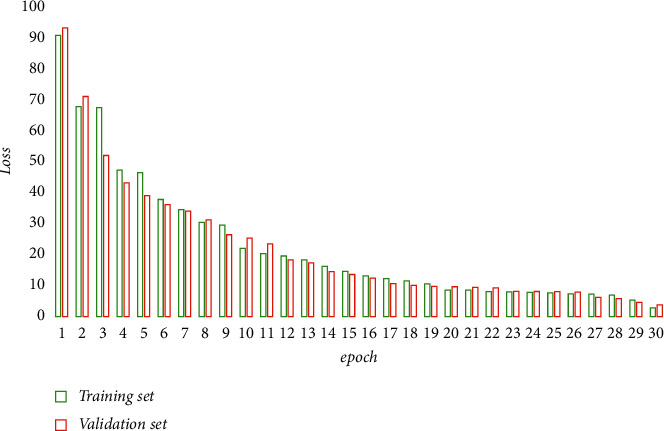
Schematic diagram of training process performance improvement.

In this study, a large multifunctional electro-hydraulic servo structural test system of 10,000 kN was used to load the steel bridge for the proposed cyclic load test. The bottom plate of the structure is connected to the base through high-strength bolts, which is cemented to the floor, and the top of the structure is connected to the two actuators through the loading beam. The vertical actuator applies axial load to the structure, the horizontal actuator applies forced displacement to the structure in the horizontal direction, and the vertical actuator follows the top of the pier to move to prevent eccentricity. The four mother plates of the structure are *A*∼*D*, and the displacement sensors are numbered 1∼5, where displacement sensors 1 and 2 are used to measure the horizontal displacement of the top of the structure and the base, respectively, and displacement sensors 3∼5 are used to monitor the loaded bridge for fatigue and fracture. The data detected by the sensors are analyzed and processed using the algorithm proposed in the text. The training process loss convergence and performance enhancement are shown in Figures 4 and 5.

### 4.2. Experimental Results

**Figure 6:**
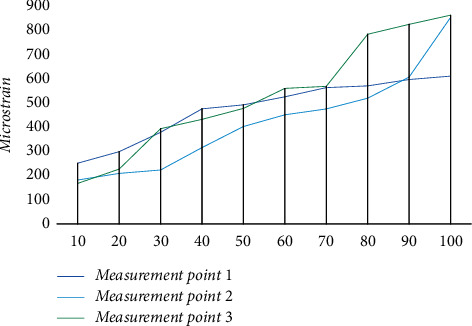
Experimental results at the first level of loading parameters.

The fatigue data under cyclic loading of the bridge steel structure experiments were obtained by using the above settings and preparations, and the fatigue curves under cyclic loading were used to complete the display of the results, considering the differences between the specimen and the time bridge, as shown in Figure 6. The fatigue curves under cyclic loading are arranged in the order of plate thickness, chord and node plate, and bridge chord angle welding. It can be seen from the images that the fatigue curves under cyclic load fluctuate more uniformly under the same load force at the measurement points. No fracture was observed when it was loaded at the first level. After multiple loading, no fatigue cracks were found at the test points under cyclic loading indicating that the bridge steel structure has good resistance to fatigue under cyclic loading at the first level of loading.

**Figure 7:**
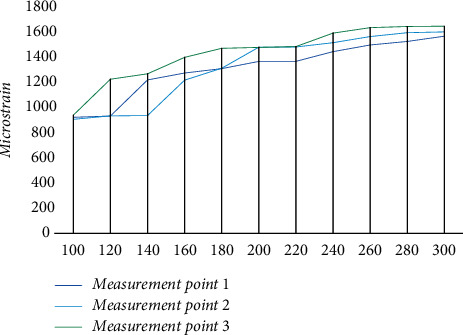
Experimental results in the second load parameter.

To verify the fatigue effect of the bridge steel structure under cyclic loading, the effect of simulated multiple transverse loads on the test points was analyzed, and the experimental results with the test points at level 2 loading are shown in Figure 7. The loading parameters were set to level 2 loading, and the corresponding experimental results were obtained as above. As the load continues to increase, part of the measurement point 1 appears to change in the form of support, reducing the concentration of the force load. In the continuous change of load values, the fatigue curve of the bridge under cyclic load resistance starts to fluctuate and affects the stability of the specimen. As the number of loading increases, the fatigue damage under cyclic loading of measurement point 1 gradually accumulates, and once the crack is formed, deformation occurs near the crack, the internal stress of the specimen is released, and the fracture phenomenon will occur at the measurement point out. Compared with measuring point 1, the curves of measuring point 2 and measuring point 3 are smoother, and their fatigue resistance under cyclic loading is better.

**Figure 8:**
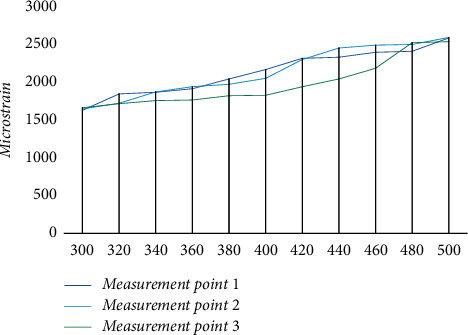
Experimental results at tertiary load parameters.

The experimental load double output is set to the limit value in the parameter, and the experimental results are obtained in the three-stage load parameter, and the experimental data are shown in Figure 8. Under the three-stage load, only one complete curve of measurement point 3 remains. According to the setting in the experimental program, the loading action on the specimen was ended when it appeared to fracture. In the conduct of this experiment, the fracture of measurement point 1 occurred when the load was increased to 300 kN, and when the load was increased to 450 kN, measurement point 2 fractured. It can be seen from the curves that when the fracture of measurement point 1 and measurement point 2 occurred, the data of some strain measurement points continued to increase after cracking, and the value of measurement point 3 appeared to retract, indicating that its measurement points should be compiled within the range of elastic deformation during the conduct of the fatigue experiment under cyclic loading.

**Figure 9:**
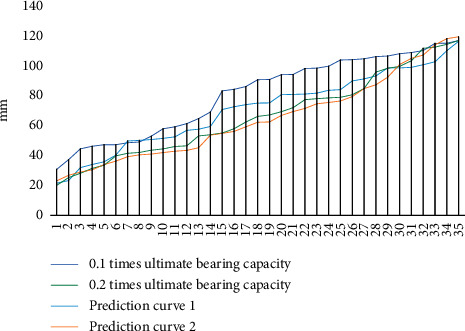
Crack length vs. number of cyclic loads.

As can be seen from Figure 9, the crack expansion is very slow at the beginning of cyclic loading, with the increase of the number of cycles, the crack is approximately linear and rapid expansion, and with the further increase of the number of cycles, the fatigue crack under cyclic loading expands sharply and becomes unstable. It is known from the above experimental results that the fatigue fracture sequence under cyclic loading under transverse load is the detailed loading part over to the smooth part. It is found that the geometry of the steel structure, the way of loading, and the distribution of the loading points directly affect the fatigue strength of the steel structure against cyclic loading. Under the combined effect of the 3 factors, the fatigue strength of steel structure under cyclic load will rise by one grade, resulting in the case of structural fracture. The fatigue performance of steel structures under cyclic loading is closely related to the welding quality. It is extremely obvious under the same parameter level test. In addition to defects arising in the welding, the surface finish of the test points has a direct effect on the fatigue strength of the steel structure under cyclic load resistance. The processing of the details of the steel structure will effectively reduce the problem of local stress concentration caused by poor welding of structural details, enhance the fatigue resistance of the bridge under cyclic loading, and prevent bridge fracture.

## 5. Conclusion

Steel bridges have been widely used in modern bridge engineering for their outstanding advantages such as light weight and high strength, large spanning capacity, easy factory fabrication, and convenient assembly construction, but the development and application of steel bridges in China have long lagged developed countries such as Europe, America, and Japan. In the current context, it is important to stand on the shoulders of giants, grasp the historical opportunity of the strategy of a strong transportation country and the vigorous promotion of steel bridges in China, introduce the latest achievements in mathematics, mechanics, theoretical analysis, test and inspection technology, processing and manufacturing technology, and artificial intelligence, and continue to make efforts in both basic theory and major engineering applications to further deepen the understanding of fatigue failure mechanisms of steel bridges under cyclic loading, and develop new methods of fatigue failure. This paper expands the understanding of fatigue failure mechanisms of steel bridges under cyclic loading and develops new theories and methods.

In this paper, we expand the research field of steel bridges according to the practical needs, propose a fatigue fracture detection model based on artificial intelligence algorithm for the fatigue fracture detection of steel bridges under cyclic loading, and establish a more complete guaranteed system for the sustainable development of fatigue fracture detection of steel bridges under cyclic loading through innovative results. In the future, we plan to start using cyclic neural networks for fatigue fracture detection of bridge steel structures under cyclic loading.

## Figures and Tables

**Table 1:** Training parameters.

Devices	Specific model
GPU	Nvidia GeForce GTX 1060 (6 GB)
CPU	Intel (R) Core i3-9100F @ 3.80 GHz
RAM	DDR4 16 GB
CUDA	CUDA 10.0
Compiler software	PyCharm Community Edition 2019
Programming languages	Python
Model framework	PyTorch

**Table 2:** Simulation of geometry and structural parameters of bridge steel structures.

Number	*B*	*D*	*h*_1_	*h*	*t*	*t*_*d*_	*t*_*s*_	*h*_*s*_	*a*	*n*	*R*_*f*_	*y*
*S*-1	0.32	0.32	1.25	1.40	7.4	7.4	7.4	39	0.16	3	0.33	0.31
*S*-2	0.20	0.20	0.65	0.80	7.4	7.4	7.4	34	0.10	2	0.31	0.28

**Table 3:** Mechanical properties of steel.

*E* (GPa)	*μ*	*o*_*u*_ (MPa)	*o*_*y*_ (MPa)	*E*_*f*_	*A* (%)
198.22	0.30	350.51	508.57	1.15	40.60

## Data Availability

The datasets used during the current study can be obtained from the corresponding author upon reasonable request.
